# How cryptic animal vectors of fungi can influence forest health in a changing climate and how to anticipate them

**DOI:** 10.1007/s00253-025-13450-0

**Published:** 2025-03-15

**Authors:** Yasin Korkmaz, Marta Bełka, Kathrin Blumenstein

**Affiliations:** 1 Faculty of Environment and Natural Resources, Chair of Pathology of Trees, University of Freiburg, Freiburg, Germany; 2 Faculty of Forestry and Wood Technology, Forest Entomology and Pathology Department, Poznań University of Life Sciences, Poznań, Poland

**Keywords:** Vector, Fungal pathogens, Spore dispersal, Interactions, Forest dynamics

## Abstract

**Abstract:**

Fungal spores are usually dispersed by wind, water, and animal vectors. Climate change is accelerating the spread of pathogens to new regions. While well-studied vectors like bark beetles and moths contribute to pathogen transmission, other, less-recognized animal species play a crucial role at different scales. Small-scale dispersers, such as mites, rodents, squirrels, and woodpeckers, facilitate fungal spread within trees or entire forest regions. On a larger scale, birds contribute significantly to long-distance fungal dispersal, potentially aiding the establishment of invasive species across continents. These vectors remain underexplored and are often overlooked in fungal disease studies and are therefore called cryptic vectors. Understanding the full range of dispersal mechanisms is critical as climate change drive shifts in species distributions and increases vector activity. Expanding monitoring and detection tools to include these hidden carriers will improve our ability to track the distribution of fungal pathogens. Integrating targeted research, innovative technologies, and collaborative efforts across disciplines and borders is essential for enhancing disease management and mitigating fungal disease’s ecological and economic impacts.

**Key points:**

*• Cryptic animal vectors play a critical role in fungal spore dispersal across forests and continents.*

*• Climate change accelerates fungal pathogen spread by altering species distributions, increasing vector activity, and facilitating long-distance dispersal.*

*• Innovative monitoring tools, like eDNA sampling and predictive modelling, are essential to uncover cryptic vector contributions and mitigate fungal disease impacts.*

## Introduction

### Mechanisms of fungal spore dispersal

Fungal sporulation involves the production of vast quantities of spores, emphasizing dispersal as a critical ecological process influencing fungal population dynamics and community structures (Urban et al. 2008). Dispersal mechanisms include wind, water, plants (e.g., driftwood or seeds), and animals. The latter two facilitated by wind and water to reach long-distance dispersal (LDD) across large areas (Tedersoo et al. 2014; Magyar et al. 2016). Examples of fungal spores with LDD via wind are *Fusarium graminearum* spores, which can disperse over 100 m; *Mycosphaerella fijiensis* spores, which can travel over 1000 m; and *Aspergillus sydowii* spores, which are capable of crossing oceans (Golan and Pringle 2017). Animals also play a key role, with invertebrates enabling short-range dispersal within localized areas, such as forest floors, while larger animals like mammals and birds contribute to LDD to over thousands of kilometers (Golan and Pringle 2017; Bielčik et al. 2019). Emerging insect pests increase these dynamics, serving as both primary and cryptic vectors of fungal pathogens. Primary vectors—such as insects—spread spores externally on their bodies, internally through their digestive systems and hemocoels, or via horizontal (environmental sources), vertical (maternal inheritance), and mixed pathways (Franco et al. 2021; Wielkopolan et al. 2021).

### Cryptic vectors: hidden agents of fungal dispersal

Beyond these well-documented mechanisms, cryptic vectors—organisms whose role in fungal dispersal is often overlooked or difficult to detect—play a critical role in fungal transmission. Unlike primary vectors, cryptic vectors do not always have an obvious association with spore dispersal, making their contributions harder to study. Cryptic carriers such as rodents, birds, and earthworms further add to the complex systems of fungal distribution patterns. These vectors can transport spores in ways that evade detection, contributing to both short- and long-distance transmission, and their activities are increasingly influenced by changing climatic conditions (Franić et al. 2023). They disperse spores through physical contact, ingestion, or excretion, often in subtle and concealed ways (Shin and Allmon 2023). Their role adds complexity to both short- and long-distance dispersal, influencing the establishment of fungal pathogens across diverse habitats (Bickford et al. 2007; Gómez-Díaz et al. 2010). The impact of cryptic vectors is particularly relevant in the context of climate change, which alters their distribution, diversity, and abundance. For example, rising temperatures and habitat shifts may expand the ranges of certain cryptic species, inadvertently increasing their role in fungal pathogen spread (Singh et al. 2023).

**Fig. 1:**
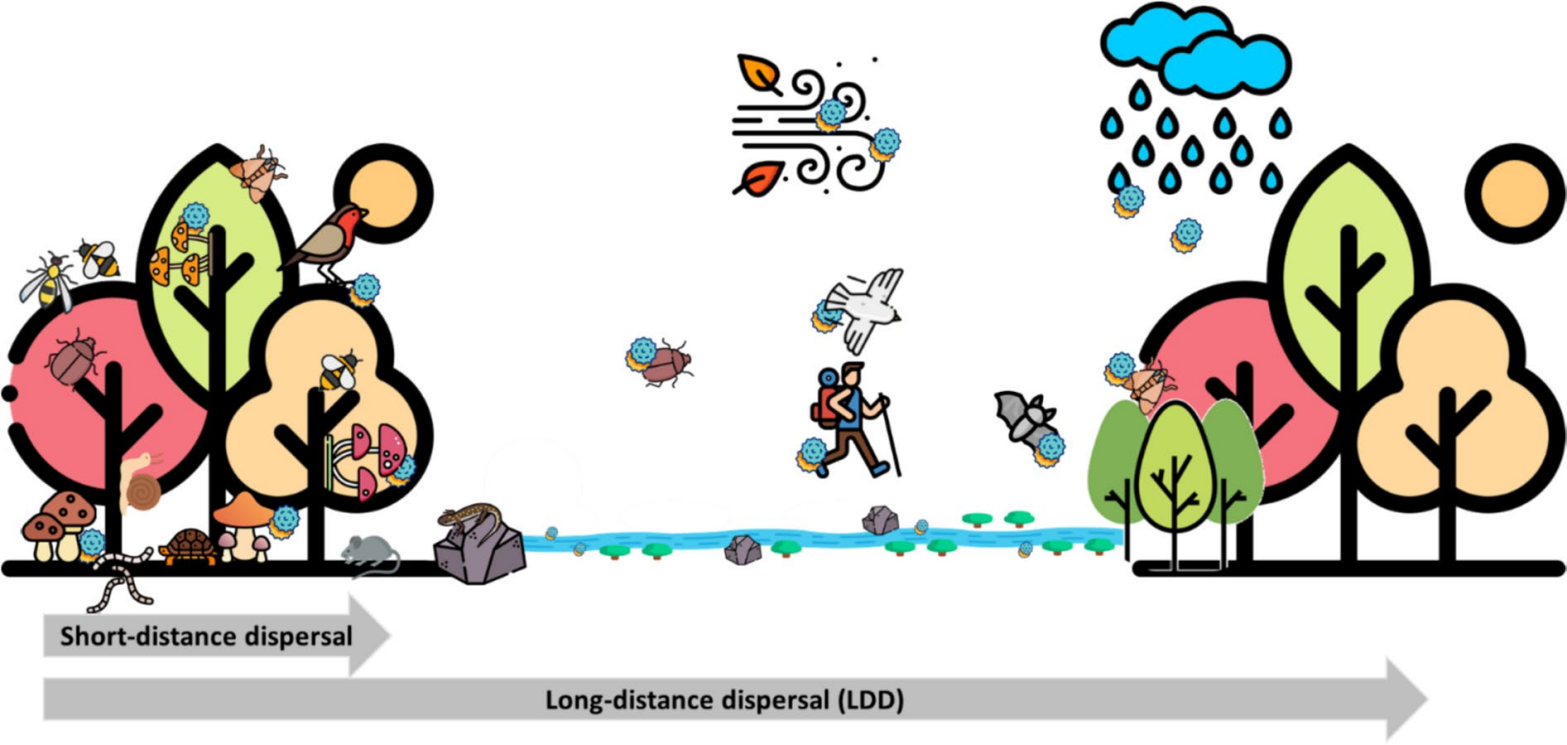
Fungal dispersal in forest stands: short- and long-distance transport mediated by animals, wind, and water (graphical elements designed by Freepik (www.Freepik.com))

Ignoring these hidden agents can result in an incomplete understanding and management of fungal diseases, emphasizing the need for targeted research (Kurtenbach et al. 2002). Expanding knowledge of cryptic vectors and their interactions with traditional dispersal mechanisms will enable the development of more effective fungal disease prevention and control strategies, thereby safeguarding ecosystems from disease outbreaks (Fig. 1).

### The growing threat of fungal tree diseases in a changing climate

Fungal diseases are increasingly threatening biodiversity and ecosystem stability, with climate change exacerbating this challenge (Lahlali et al. 2024). Rising global temperatures and altered precipitation patterns facilitate the proliferation and spread of fungal pathogens (Burdon and Zhan 2020; Franić et al. 2023). These shifts enable pathogens to expand their geographic ranges, exposing previously unaffected populations and ecosystems to significant risks (Baker et al. 2022). Climate change disrupts host–pathogen dynamics and fosters novel infectious diseases by altering habitats and species distributions (Singh et al. 2020; Chen et al. 2024). For example, changing temperature and precipitation patterns remove overwintering barriers for pathogens, allowing them to thrive in new regions and infect a broader range of hosts (Velásquez et al. 2018; Singh et al. 2023). Additionally, warmer winters improve survival rates for pathogens and invasive insect pests, accelerating their life cycles and enabling multiple generations during growing seasons (Singh et al. 2023). The evolution of more virulent fungal strains is another pressing issue, as warmer temperatures promote pathogen adaptation to new climates, potentially overwhelming plant defenses (Cohen and Leach 2020; Mesny et al. 2024). These threats are compounded by prolonged growing seasons and expanded pathogen ranges, intensifying plant vulnerability and threatening forests and agricultural systems. The globalization of trade complicates control efforts, as pathogens spread through seeds, timber, and nursery stock, encountering hosts with limited resistance in new regions (Franić et al. 2024). Changes in fungal community composition also exert selective pressures on spore morphology, influencing their discharge, transport, and deposition across niches, significantly impacting plant fitness and ecosystem functioning (Peay and Bruns 2014; Calhim et al. 2018). Addressing these cascading effects requires an integrated understanding of fungal disease dynamics and the interactions between pathogens, vectors, hosts, and environmental changes to protect global ecosystems.

## Material and methods

A structured literature search was conducted to compile this mini-review, utilizing several scientific databases, including Google Scholar, Web of Science, ScienceDirect, SpringerLink, JSTOR, PubMed, CABI, and EPPO. For each thematic section (e.g., fungal pathogen dispersal mechanisms and vector biology), separate searches were performed using targeted keywords. The primary keywords included *fungi* (with a focus on plant pathogenic species), *vector*, *plants* (with an emphasis on trees and forests), and their interactions. Secondary keywords included *dispersal*, *vector-mediated spread*, *pathogen dynamics*, and *vector ecology*, all combined with Boolean operators (AND, OR) to ensure a comprehensive retrieval of relevant studies. Recent peer-reviewed publications were prioritized to reflect the most up-to-date understanding of fungal pathogen dispersal and vector dynamics, while also incorporating seminal studies that remain foundational to the field. Articles were selected based on their methodological rigor, inclusion of empirical data or field-based case studies, and relevance to the role of vectors in pathogen spread. Where applicable, in-depth reviews on specific subsections are referenced, directing to more specialized discussions beyond the scope of this mini-review.

## Key agents in fungal pathogen dispersal

### Mechanisms by invertebrates

#### Passive transport through physical attachment

Invertebrates serve as efficient vectors for fungal spore dispersal by transporting spores across diverse environments. One primary dispersal method involves spores attaching to the invertebrates’ integuments or external body surfaces (Koch and Aime 2018; Kitabayashi et al. 2022; Santamaria et al. 2023). The exoskeletons of insects and other invertebrates provide a large surface area for spore adhesion, allowing many spores to be carried simultaneously (Joseph and Keyhani 2021). This method is particularly effective because it leverages the mobility of invertebrates to transport spores over considerable distances (Golan and Pringle 2017; Gill et al. 2022). As these invertebrates move through various habitats, they inadvertently facilitate the spread of fungal spores, enhancing the reach and impact of fungal pathogens.

- *Bark beetles* play a critical role in the spread of fungal pathogens due to their mutualistic relationships with various fungi. These beetles carry fungal spores on their exoskeletons while feeding and nesting in tree bark (Six and Wingfield 2011). One of the most well-known examples is Dutch Elm Disease (DED), caused by *Ophiostoma novo-ulmi* and spread by elm bark beetles (*Scolytus* spp.), which has devastated elm populations in North America and Europe (Webber et al. 1984; Jürisoo et al. 2021). Similarly, laurel wilt disease, caused by *Harringtonia lauricola* and vectored by the Redbay ambrosia beetle (*Xyleborus glabratus*), has led to severe avocado tree mortality (Conover et al. 2024). The mountain pine beetle (*Dendroctonus ponderosae*) and the blue stain fungus (*Grosmannia clavigera*) have caused widespread pine forest mortality in North America (Reed et al. 2018). Additionally, oak wilt, caused by *Bretziella fagacearum*, is spread by *Nitidulidae* beetles and oak bark beetles (*Pseudopityophthorus* spp.) (EPPO 2024). Black stain root disease, caused by *Leptographium wageneri*, is vectored by *Hylastes nigrinus* and long-snouted weevils (Schowalter 2018). Bark beetles are also involved in the spread of *Armillaria* spp., a genus of fungi that causes root rot in various tree species, which can lead to increased tree mortality, particularly in stressed or weakened trees (Jakuš 2001). Some fungi, like *Raffaelea* spp., produce chemical attractants to guide beetles, facilitating the spread of spores (Reed et al. 2018; Kandasamy et al. 2023). Bark beetles can also be associated with *Phytophthora ramorum*, the pathogen responsible for sudden oak death. Research has shown that trees infected with *P. ramorum* attract bark beetles, which can further intensify the disease’s spread by serving as vectors for the pathogen (McPherson et al. 2008). Similarly, the mango bark beetle (*Hypocryphalus mangiferae*) is associated with *Neonectria* spp., which are known to cause diseases in mango trees, illustrating how bark beetles can vector pathogens that affect economically important crops, leading to significant agricultural losses (Lee et al. 2023).
- *Honey bees and flies*: While foraging for nectar or feeding on decaying organic matter, honey bees can transport spores of fungi like *Sclerotinia sclerotiorum* and *Verticillium albo-atrum* across diverse habitats (Card et al. 2007; Sazima and Sazima 2022). Flies are attracted to fungi especially from the odor-producing *Phallaceae* family, such as stinkhorns, which rely on flies for spore transmission that are transported as the flies land on decaying organic material (Sazima and Sazima 2022). Additionally, both bees and flies become contaminated through contact with oozing cankers and subsequently spread spores to flowers, facilitating infections that often affect leaves and twigs via wounds (Mitchell 2004; Agrios 2008).
- *Mites* transport spores on their exoskeletons as they traverse plant surfaces (Roets et al. 2011). Notable examples of fungal pathogens spread by mites include *Alternaria alternata* and powdery mildews like *Erysiphe alphitoides* (Hubert et al. 2003; Tack et al. 2012). In the case of mango malformation disease caused by *Fusarium moniliforme*, the eriophyid mite *Aceria mangifera* is a primary vector. Also, mites have been implicated in the spread of certain pine rust diseases (Agrios 2008). Additionally, mites can influence the dynamics of insect-fungal mutualisms by enhancing fungal transmission, reproduction, and survival. They may achieve this by feeding on antagonistic fungi or promoting microbes that reduce insect-pathogenic fungi (Hofstetter and Moser 2014).

#### Active transport through ingestion and excretion

Invertebrates actively transport fungal spores when ingested during feeding and later excreted in new locations (Kitabayashi et al. 2022). This process has been demonstrated to improve germination rates of spores, facilitating the establishment of fungi in new environments. Additionally, fungal propagules and hyphae can survive passage through the digestive systems of various invertebrates (Vašutová et al. 2019).

- *Moths*: Moths play an active role in transporting fungal spores during feeding, pollination, and egg-laying, making them essential vectors in both natural ecosystems and agricultural systems. While some species facilitate local dispersal, others contribute to long-distance transmission. For example, young spongy moth (*Lymantria dispar*) caterpillars preferentially feed on *Populus nigra* foliage infected by *Melampsora larici-populina*, selectively consuming fungal spores and potentially aiding their spread (Eberl et al. 2020). Similarly, moths have been implicated in the transmission of *Monilinia fructicola*, the causal agent of brown rot in peaches and cherries, as well as *Botrytis cinerea*, with spores transferred during feeding and egg-laying activities (Mondy et al. 1998; Holb 2013). In addition to these localized interactions, certain migratory moth species serve as primary vectors of fungal pathogens over vast distances. The corn earworm moth (*Helicoverpa zea*) has been documented carrying viable spores of *Claviceps africana* internally for several days, enabling its spread from endemic regions like Mexico and southern Texas to previously unaffected areas in the USA (Prom and López 2004). Likewise, *H. zea*, and other migratory moths have been linked to the long-range dispersal of *Claviceps paspali* in Japan, demonstrating how moth migration patterns can shape fungal pathogen dynamics across both natural and agricultural landscapes (Sugiura and Yamazaki 2006).
- *Slugs* ingest fungal fruiting bodies and excrete viable spores, which can germinate and colonize new areas. This mechanism facilitates the spread of fungi from the genus *Pleurotus*, *Armillaria*, and *Gymnopilus* in forest ecosystems (Kitabayashi et al. 2022). Field experiments indicate that slugs can travel up considerable distances within hours by moving across the ground, litter layers, wood debris, and tree trunks. These movements enable slugs to transport ectomycorrhizal, saprophytic, and wood-decaying fungi spores to suitable sites for colonization (Kitabayashi et al. 2022). Additionally, slugs (*Lehmannia marginata*) have been observed feeding on chestnut blight cankers and acting as vectors for the chestnut blight disease (Turchetti and Chelazzi 1984).
- *Earthworms* ingest spores and organic matter and excrete them in nutrient-rich casts, enhancing fungal viability and soil fungal diversity (Chaudhary et al. 2022). Studies show that earthworms and their casts contain higher amount of spores and infective propagules than surrounding soil (Vašutová et al. 2019; Edwards and Arancon 2022). Fungal spores can survive through the earthworm digestive system and remain viable in dried casts (Vašutová et al. 2019). Earthworms influence spore dispersal patterns through direct and indirect grazing and may selectively digest certain fungi, shaping fungal communities. In a study by Montecchio et al. (2015), the plant pathogens *Fusarium* and *Verticillium* were found in seedlings along forest edges, passively vectored by earthworms.
- *Aphids, whiteflies, and psyllids*: By feeding on plant sap, they create wounds that serve as entry points for fungi and excrete honeydew, a sugary substance that promotes the growth of sooty mold (*Capnodium* spp.), reducing photosynthesis and overall plant health (Magyar et al. 2016; Josephrajkumar et al. 2023). Aphids, such as the woolly aphid (*Eriosoma lanigerum*), transmit *Neofabrea perennans*, the causal agent of perennial apple canker (Agrios 2008). Whiteflies similarly produce honeydew, encouraging sooty mold growth and impairing plant productivity (Josephrajkumar et al. 2023). Additionally, aphids and whiteflies can carry fungal spores like *Alternaria alternata*, facilitating their spread between plants (Monzo and Stansly 2017).
- *Wasps* transport fungal spores either through their digestive systems or on their bodies while foraging for nectar or decaying organic matter (Sazima and Sazima 2022). One example of a wasp species that aids in the dispersal of fungal pathogens through direct interactions is the European paper wasp (*Polistes dominula*). Research indicates that the nests of *P. dominula* can harbor a variety of fungal species, including plant pathogens such as *Alternaria*, *Cladosporium*, *Corallomycetella*, *Penicillium*, *Phoma*, and *Pseudozyma* spp. These fungi can contribute to the spread of fungal diseases in the environment (Yamoah et al. 2008). Another notable example is the female woodwasp (*Sirex noctilio*), which carries the white-rot fungus *Amylostereum areolatum* in specialized structures called mycangia and inoculates the fungus into trees during egg-laying (Slippers et al. 2011). Additionally, in birch constriction disease, the apical birch woodwasp (*Pseudoxiphydria betulae*) feeds on birch shoots, causing constrictions that lead to leaf withering and death. Remarkably, 92% of these constrictions are also infected with the anthracnose fungus *Melanconium bicolor* (Agrios 2008). Some shelf fungi, such as *Cerrena unicolor*, have specialized relationships with wood-boring wasps, where the wasps transmit the fungal inoculum into the wood through their ovipositors during egg-laying (Elliott et al. 2022b).

#### Symbiotic relationships between fungi and invertebrates

Invertebrates often form specialized mutualistic relationships with fungi, enhancing spore dispersal and influencing ecosystem dynamics. Mutualism is a symbiosis where both partners benefit, leading to significant fitness gains for one or both. These partnerships can manifest as resource-resource exchanges (e.g., nutrients), service-resource exchanges (e.g., dispersal for nourishment), or service-service exchanges (e.g., protection for dispersal) (Elkhateeb 2021). Such interactions promote fungal propagation and support the survival and ecological success of the invertebrates involved, thereby shaping broader ecosystem functions.

- *Bark beetles and ophiostomatoid fungi*: Bark beetles facilitate the transport of ophiostomatoid fungi using specialized structures called mycangia, ensuring the viability and effective dispersal of fungal spores (Mayers et al. 2022). These mutualistic relationships contribute to significant tree diseases and blue-stain fungus infestations (Six and Wingfield 2011). For example, blue-stain fungi are carried by bark beetles like *Dendroctonus ponderosae* and *Ips pini*, which create wounds that allow fungal penetration. In turn, the fungi lower the tree’s water content and enhance the microenvironment for the beetles’ developing larvae. This interaction exemplifies mutualistic symbiosis, where the fungi and insects benefit (Agrios 2008).
- *Ambrosia beetles and fungal farming*: Ambrosia beetles, a group of fungus-growing weevils, engage in specialized mutualistic relationships with fungi, particularly those in the orders *Microascales* and *Ophiostomatales* (Hulcr and Stelinski 2017). These beetles cultivate fungi, such as *Raffaelea* spp., in galleries within wood, a behavior known as fungal farming. The fungi, called ambrosia fungi, are fully domesticated and cannot survive independently of their beetle hosts (Mayers et al. 2022). In return, the fungi provide a primary food source for beetle larvae, ensuring the survival and growth of the beetle population (Diehl et al. 2022). This mutualistic interaction has significant ecological impacts, mainly when invasive beetle species contribute to crop diseases.
- *Termites and wood-decaying fungi*: Termites, particularly those in the subfamily *Macrotermitinae*, engage in a specialized mutualistic relationship with fungi in the genus *Termitomyces* (Vega and Blackwell 2005). These termites cultivate *Termitomyces* in their nests, forming fungus gardens that break down lignocellulose in plant material. This relationship enables termites to process woody biomass efficiently and provides a stable habitat for fungal growth (Chiu et al. 2023). *Termitomyces* fungi play a crucial role in decomposing plant biomass, especially lignin-enriched substrates, by depolymerizing complex phenolic polymers (Schalk et al. 2021). In return, termites benefit from a nutritionally enriched and dependable food source (Schmidt et al. 2024).

### Vertebrates as intermediate and long-distance dispersers

Vertebrates contribute to fungal pathogen dispersal by moving spores between distant habitats or within ecosystems. Mammals, birds, and reptiles play substantial roles in spreading fungal spores directly or indirectly through attachment to fur, feathers, or scales.

#### Mammals as fungal spore dispersers: local and long-distance agents

Interactions between soilborne fungi, woody plants, and mycophagous mammals form critical mutualistic symbioses that drive fungal spore dispersal. Fungi that produce underground fruiting bodies, such as hypogeous fungi, depend on animals for dispersal, as active spore discharge and aerial transport are impossible below ground (Komur et al. 2021).

- *Rodents and small mammals*: Rodents and other small mammals are key dispersers of hypogeous fungi. By consuming truffles and other fungal sporocarps, they ingest spores and excrete them in new locations, promoting fungal colonization and enhancing local fungal diversity (Stephens and Rowe 2020). For example, in Eucalyptus-dominated forests, rodents primarily disperse ectomycorrhizal fungi, whereas in wetter rainforest habitats, they disperse vesicular–arbuscular fungi (Elliott et al. 2022a).
- *Squirrels*: The Japanese squirrel (*Sciurus lis*) consumes fungi such as *Amanita muscaria* and helps disperse spores (Suetsugu and Gomi 2021). North American squirrel species, including the northern flying squirrel, Douglas’ squirrel, and Townsend’s squirrel, are particularly effective dispersers of truffle spores (Duncan and Carey 2004).
- *Digging mammals*: In Australia, digging mammals such as the quenda (*Isoodon fusciventer*) forage for fungi, disturbing the soil and facilitating fungal growth and spore distribution (Hopkins et al. 2024). These mammals are also implicated in the spread of fungal pathogens in urban environments (Komur et al. 2021).
- *Bats*: Though less recognized, bats contribute to long-distance fungal spore dispersal. Their frequent movement between caves, forests, croplands, and human settlements enables them to carry spores on their bodies. This behavior aids the spread of plant pathogens like *Aspergillus flavus*, *Fusarium incarnatum*, and *Neocosmospora* spp., which threaten crops and pose risks to human and animal health (Karunarathna et al. 2023).

#### Reptiles as unrecognized vectors

Several reptilian species engage in mycophagy (fungus consumption), playing a significant role in fungal spore dispersal. Spores can be transported internally through ingestion and excretion or externally on skin and scales and mucous membranes. Box turtles (*Terrapene carolina*) are among the most commonly reported mycophagous reptiles, known to consume toxic fungi like Amanita species (Elliott et al. 2019). By ingesting fungal fruiting bodies and excreting spores, they can contribute to dispersing plant and animal pathogens such as *Aspergillus* sp. (Jones et al. 2007). Additionally, reptilian species have been shown to harbor common fungal plant pathogens, including *Aspergillus* sp., *Mucor* sp., *Penicillium* sp., and *Geotrichum* sp. (Schumacher 2003). Other reptiles also facilitate spore dispersal. Skinks, such as the land mullet skink (*Egernia major*), dig up and consume hypogeous fungi. In contrast, blue-tongue skinks (*Tiliqua scincoides*) feed on the fruiting bodies of the stinkhorn fungus (*Aseroe rubra*) (Elliott 2019). Snakes may serve as effective secondary vectors. Both constrictors and venomous snakes consume their prey as whole, including rodents and small marsupials known to eat various fungi (Nuske et al. 2017). This behavior enables the indirect dispersal of fungal spores through snake feces. Reptiles’ diverse fungal diets, which include toadstools, shelf fungi, truffles, and puffballs (Elliott et al. 2019), highlight their underappreciated role in fungal dispersal and ecosystem health.

#### Birds as long-distance dispersers (LDDs) of fungal spores

Birds, particularly migratory species, are highly effective at LDD, which is crucial in spreading fungi across biogeographical boundaries. They transport spores through two primary mechanisms: ingestion (endozoochory) and external attachment (epizoochory). Migratory patterns, especially in the northern hemisphere’s temperate regions, concentrate dispersal along specific corridors, facilitating the spread of beneficial and pathogenic fungi (Altizer et al. 2011; Viana et al. 2016). For instance, woodpeckers in Finland, which migrate to Eurasian countries, have been shown to carry *Fusarium* species on their feathers, potentially introducing these pathogens to new regions (Johansson et al. 2021). Similarly, flower-visiting birds play a critical role in fungal dispersal. By feeding on flowers, they co-transport fungal spores and pollen grains, increasing the likelihood that spores are deposited in environments conducive to their development, such as on other flowers (da Silva et al. 2016; Viana et al. 2016). This co-dispersion enhances fungal colonization opportunities in suitable habitats. A study by Alfonzo et al. (2013) isolated 2337 filamentous fungi from 216 migrating Mediterranean birds, identifying *Cladosporium cladosporioides*, *Alternaria alternata*, and *Aspergillus niger* as the most abundant species. Additionally, an experiment in Poland demonstrated that birds could transmit *Phytophthora* species, with transmission frequency varying by bird species such as siskins, greenfinches, and tits (Malewski et al. 2019). The facilitated dispersal of fungal (or oomycete, in case of *Phytophthora* spp.) pathogens through bird migration has significant ecological and economic consequences. It enables fungi to spread across continents, potentially leading to disease outbreaks and biological invasions (Altizer et al. 2011; Green 2016). By dispersing fungal spores across diverse habitats, birds enhance fungal diversity and ecosystem functioning and contribute to the spread of pathogenic fungi, influencing natural and human-modified ecosystems.

#### Human activity as a major driver of fungal pathogen spread

Human activity is perhaps the most significant driver of fungal pathogen spread. Global trade, travel, and deforestation have facilitated the rapid movement of fungal pathogens across the globe. Fungal spores are often transported on vehicles, footwear, or forestry equipment in contaminated soil. The international trade in ornamental plants and seeds also provides pathways for fungi to enter new ecosystems, sometimes introducing invasive pathogens. Human-mediated dispersal accelerates the spread of existing pathogens and fosters the emergence of new, hybrid strains. For example, plant disease epidemics caused by introduced fungal pathogens such as *Cryphonectria parasitica* (chestnut blight), *Ophiostoma ulmi* and *Ophiostoma novo-ulmi* (Dutch elm disease), *Ceratocystis platani* (Plane canker), and *Cronartium ribicola* (white pine blister rust) illustrate the impact of human-mediated long-distance dispersal (LDD) (Tsopelas et al. 2017; Golan and Pringle 2017). Moreover, climate change exacerbates these risks by altering fungal life cycles, allowing pathogens to thrive in regions where they were previously unable to survive (Lahlali et al. 2024). Additionally, man-made vectors such as shipping, agriculture, and aquaculture boost the dispersal potential of fungal species, enabling transoceanic and transcontinental transport and range expansions well outside their natural capabilities (Pérez-Portela et al. 2013).

## Monitoring and analysis of cryptic vectors

### Importance of tracking

The importance of tracking cryptic vectors lies in the substantial knowledge gap regarding their specific contributions to forest health and fungal pathogen dynamics. Unlike well-documented dispersal mechanisms, cryptic vectors remain largely unmeasured regarding their global impact on forest ecosystems. Their role in transporting fungal spores from infected to uninfected trees is particularly concerning, as these subtle interactions may worsen tree health decline and amplify the spread of pathogens. Despite this potential, their contributions are often overlooked or underestimated. This oversight is especially critical in climate change, accelerating the dispersal and establishment of pathogens in new regions. Rising temperatures, altered precipitation patterns, and habitat shifts create conditions conducive to fungal proliferation, making cryptic vectors key players in pathogen spread. As forests worldwide face increasing threats from fungal diseases, understanding and quantifying the role of these hidden carriers becomes essential. Without targeted research, their activity’s ecological and economic ramifications will remain unaddressed, leaving forests more vulnerable to future challenges.

### Innovative monitoring techniques and broader strategies

#### Passive monitoring and behavioral observations

Passive monitoring is essential for studying cryptic vectors and fungal pathogens without disturbing their natural behaviors. This approach gathers authentic data on vector movements, feeding patterns, and fungal dispersal routes. Fecal analysis provides critical insights into vector-pathogen interactions by identifying viable fungal spores in the feces of reptiles, rodents, and slugs. Many rodents’ small home ranges and rapid digestion rates make fecal analysis reliable for detecting fungal sporocarps in specific habitats (Bradshaw et al. 2022). Understanding vector behavior, such as locomotion, feeding, and dispersal, aids in identifying vector-pathogen relationships (Hoy 2019). For instance, bark beetles use aggregation pheromones to attract conspecifics, facilitating fungal transmission (Frühbrodt et al. 2024; Bracalini et al. 2024). Monitoring flying insects like moths and wasps is challenging due to their small size and nocturnal activity (Kirkeby et al. 2016). That is why field-based methods provide scalable tools for vector documentation:

- *Sticky traps* capture flying insects such as moths, aphids, and leaf miners. Yellow sticky traps are particularly efficient for pest monitoring (Ramasamy and Ravishankar 2018).
- *Pitfall traps* efficiently collect ground-dwelling invertebrates like beetles and slugs for long-term sampling (Bertoia et al. 2023).
- *Motion-activated cameras* monitor larger mammals like rodents, bats, and reptiles, minimizing handling stress and enabling continuous, non-invasive observation (Hopkins et al. 2024).

#### Broader surveillance strategies

Comprehensive surveillance strategies integrate data across ecosystems, including forests, agricultural landscapes, and urban environments, to track cryptic vectors and pathogens on a large scale. Collaboration with citizen science programs and community-based monitoring initiatives enhances cost-effective data collection, increases public awareness, and enables researchers to gather long-term datasets with broad spatial coverage (Gupta et al. 2022; Sgroi et al. 2023). For instance, the *Ceratocystis platani* and *Xylella fastidiosa* Protected Zone Status Survey (2016) used trained personnel to monitor tree pathogens across London (Gupta et al. 2022). The TreeSnap mobile app (https://treesnap.org/) connects citizen scientists with researchers to track tree pests and disease distribution. It helps identify invasive threats like the emerald ash borer on ashes and Dutch elm disease on American elms. TreeSnap also assists in locating healthy or resistant trees for breeding programs, supporting forest restoration efforts (Crocker et al. 2020). Early detection and surveillance mechanisms enable rapid responses, such as eradication, containment, or control, to mitigate pest and disease impacts (FAO 2018). Additionally, international collaborations are essential for tracking migratory species, such as birds and bats, which can disperse fungal spores across continents. Understanding movement patterns, including migratory connectivity between breeding, stopover, and wintering sites, helps identify limiting factors and informs conservation efforts (Marra et al. 2011).

#### Advanced technologies for ecological monitoring

Innovative technologies are transforming the monitoring of cryptic vectors and their interactions with fungi by enabling non-invasive and precise data collection. Environmental DNA (eDNA) sampling detects fungal spores and vector DNA from soil, water, and air, identifying species even without direct observation. This method is crucial for biosecurity and invasion biology, helping track biological invasions from early detection to eradication confirmation (Bell et al. 2024). Remote sensing technologies, such as satellite imagery and drones, monitor environmental changes and identify potential vector habitats, including areas affected by tree diseases or deforestation. These techniques provide unbiased, quantitative data across various spatial and temporal scales (Cook and Hockings 2011). The Copernicus Land Monitoring Service (CLMS) (https://www.copernicus.eu/en) offers high-resolution, open-access datasets on land cover and land use, supporting forest health monitoring (EEA & DG JRC). Tracking technologies enable real-time vector monitoring, including RFID tags, GPS devices, and automated camera systems. Radio-telemetry effectively tracks small organisms like insects, reptiles, birds, and bats with high temporal and spatial precision, eliminating the need for recapture (Kays et al. 2015). The Motus Wildlife Tracking System (https://motus.org) uses automated radio-telemetry arrays to study small flying organisms’ movements across regional and hemispheric scales (Taylor et al. 2017). GPS data loggers provide accurate position estimates (± 30 m), tracking long-distance movements and delineating migratory connectivity (Hallworth and Marra 2015).

### Predictive modelling for disease management and mitigation

Predictive modelling is a crucial tool for forecasting the spread of fungal pathogens. These models integrate data on vector behavior, environmental conditions, and fungal biology to predict outbreaks and identify high-risk areas, ultimately aiding in the mitigation of disease spread. While machine learning (ML) has gained significant attention, it is important to recognize that predictive modelling extends far beyond ML, incorporating traditional and established methodologies that continue to play vital roles in this field. Machine learning has proven beneficial for building computational models from large datasets, allowing systems to identify patterns and predict outcomes (Baştanlar and Özuysal 2014; Handelman et al. 2018). For example, automated disease detection systems use field sensors to collect plant images, which ML algorithms then analyze to determine if leaves are healthy or diseased (Jafar et al. 2024). Similarly, species distribution modelling (SDM) allows for the estimation of geographic ranges of vectors and pathogens, predicting how environmental variables influence their spread (Yoon and Lee 2023). Climate-based models predict how warming temperatures could expand the range of vectors like bark beetles, leading to increased fungal pathogen spread. SDM has been applied to insect-borne diseases such as *Xylella fastidiosa*, spread by xylem-feeding vectors (Rossi and Rasplus 2023). Despite the centrality of machine learning in some aspects of disease prediction, many predictive models in ecology and epidemiology do not rely on ML. For instance, Bayesian species distribution models have been used to predict wildlife distribution without ML, showing that robust predictions can be made with presence-only and presence-absence data (Morera‐Pujol et al. 2022). In demographic modelling, studies like that of Osawa et al. use virtual ecological models based on differential equations to predict movements in animal populations, such as sika deer, without the need for machine learning techniques (Osawa et al. 2023). Similarly, in epidemiology, differential equations and compartmental models are commonly used to predict disease transmission, as seen in malaria and Zika virus modelling, highlighting the flexibility of traditional approaches in the face of complex dynamics (Tesla et al. 2018). Additionally, integrating climate data into predictive models, as demonstrated by Fick and Hijmans’ research on generalized additive models (GAMs), further emphasizes the effectiveness of traditional statistical techniques in predicting species distributions based on environmental factors (Elith et al. 2010; Fick and Hijmans 2017). Real-time monitoring and adaptive management strategies, guided by these models, allow for targeted fungicide and insecticide applications that enhance plant health while reducing the risk of resistance in fungi and insects (Corkley et al. 2022).

## Conclusion

Cryptic vectors, including various invertebrates and vertebrates, play a critical yet often overlooked role in the dispersal of fungal pathogens, particularly in climate change. These vectors facilitate both local and LDD of fungal spores, contributing to the spread of diseases that threaten biodiversity, forest health, and agriculture. While traditionally understudied, cryptic vectors such as insects, rodents, and birds significantly impact pathogen dynamics by dispersing spores across landscapes. Climate change worsens this issue by increasing vector activity through rising temperatures, altered precipitation patterns, and shifting species distributions, allowing pathogens to spread to new regions. Addressing the challenges created by these vectors requires a comprehensive approach that integrates advanced monitoring techniques and collaborative surveillance efforts. eDNA sampling and predictive modelling enhance early detection and enable proactive management strategies. Collaborative networks, such as those involving citizen scientists, expand data collection capacity and support large-scale monitoring. Recognizing the importance of cryptic vectors is essential for developing effective disease management and mitigation practices. Expanding research to include these hidden agents can improve our ability to track fungal pathogen movement and identify high-risk areas. This approach will lead to more informed strategies for controlling the spread of fungal diseases, ultimately helping to protect ecosystems, forests, and agricultural systems from the growing threat of outbreaks. Addressing these challenges in the face of climate change is crucial to safeguarding biodiversity and ecosystem health.

### Guidance for further reading

*Mechanisms of fungal spore dispersal*: For a detailed exploration of fungal spore dispersal, see Golan and Pringle (2017).

*Cryptic vectors*: For insights into the role of hidden agents in fungal dispersal, refer to Peay et al. (2012).

*The growing threat of fungal tree diseases in a changing climate*: To understand the impact of climate change on fungal diseases, see Wang et al. (2021).

*Key agents in fungal pathogen dispersal*: For a review of invertebrate dispersal mechanisms, including passive and active transport and for information on symbiotic relationships between fungi and invertebrates, refer to Santamaria et al. (2023).

*Vertebrates as intermediate and long-distance dispersers*: For an overview of mammalian dispersal of fungal spores, see Elliott et al. (2022b). For insights into reptiles as fungal spore dispersers, see Elliott (2019). For information on birds as long-distance dispersers, refer to da Silva et al. (2016).

*Human activity as a major driver of fungal pathogen spread*: For an exploration of how human actions contribute to fungal pathogen spread, see Golan and Pringle (2017).

*Monitoring and analysis of cryptic vectors*: For innovative strategies in tracking cryptic vectors, see Bell et al. (2024). For a discussion on predictive modeling for fungal disease management, refer to Osawa et al. (2023).
